# WiFi RSS and RTT Indoor Positioning with Graph Temporal Convolution Network

**DOI:** 10.3390/s25247622

**Published:** 2025-12-16

**Authors:** Lila Rana, Aayush Dulal

**Affiliations:** 1 Department of Electrical and Computer Engineering, The University of Texas at Dallas, Richardson, TX 75080, USA; 2 Department of Mechanical Engineering, The University of Texas at Dallas, Richardson, TX 75080, USA; aayush.dulal@utdallas.edu

**Keywords:** wifi indoor positioning, wifi RSS and RTT, temporal convolution network, graph convolution network

## Abstract

Indoor positioning using commodity WiFi has gained significant attention; however, achieving sub-meter accuracy across diverse layouts remains challenging due to multipath fading and Non-Line-Of-Sight (NLOS) effects. In this work, we propose a hybrid Graph–Temporal Convolutional Network (GTCN) model that incorporates Access Point (AP) geometry through graph convolutions while capturing temporal signal dynamics via dilated temporal convolutional networks. The proposed model adaptively learns per-AP importance using a lightweight gating mechanism and jointly exploits WiFi Received Signal Strength (RSS) and Round-Trip Time (RTT) features for enhanced robustness. The model is evaluated across four experimental areas such as lecture theatre, office, corridor, and building floor covering areas from 15 m × 14.5 m to 92 m × 15 m. We further analyze the sensitivity of the model to AP density under both LOS and NLOS conditions, demonstrating that positioning accuracy systematically improves with denser AP deployment, especially in large-scale mixed environments. Despite its high accuracy, the proposed GTCN remains computationally lightweight, requiring fewer than $10^{5}$ trainable parameters and only tens of MFLOPs per inference, enabling real-time operation on embedded and edge devices.

## 1. Introduction

Global Positioning System (GPS) technology [1] has become the state of the art when it comes to outdoor location estimation; in contrast, indoor positioning is a more difficult problem because of obstacles and environmental factors, both natural and human-developed. WiFi Positioning System (WPS) makes accurate indoor positioning possible by using wireless signals and access points [2,3,4,5,6,7,8,9,10,11,12]. These systems rely on the abilities of components to quantify elements such as signal strength and signal travel time. In addition, it is important for the components of the system to be economically viable and easy to deploy [13].

Most wireless indoor positioning systems can be classified as either range-based or fingerprint-based. Range-based methods rely on metrics such as the Received Signal Strength (RSS), Round-Trip Time (RTT), and Time of Arrival (ToA). Fingerprinting methods work by building a radio map of signal features, which is used to compare with new measurements to estimate the closest matching position [14]. Proximity-based methods have also been proposed due to their simplicity; however, accuracy decreases during operation [15]. Use of Bluetooth [16], ultrasonic signals [17], radio frequency [18], and computer vision [19] has also been studied, but each has its disadvantages. The use of Bluetooth is limited by coverage problems due to its inability to scale to larger areas, and ultrasonic-based devices have a high production and deployment cost. In contrast, RFID-based technology is cheaper, but like Bluetooth lacks coverage. ZigBee technology [20] is inexpensive and power consumption is low in comparison to other technologies, but it has problems maintaining stability during operation. Use of computer vision has become extremely common in numerous applications, including indoor positioning. Despite its popularity, computer vision-based devices and algorithms leverage the camera feed, which is prone to disturbance and obstacles. Because of these limitations of different technologies, WiFi-based solutions have generated interest in the research and development of indoor positioning systems.

Using a fingerprinting-based method, the problem of indoor positioning can be broken down into two phases: the offline phase and the online phase. The offline phase is when signal fingerprints are obtained at known positions to build a dataset; during online query, the Received Signal Strength Indicator (RSSI) vector [2,4,5,8] is measured and compared with entries in the database from the phase before. For simplicity, this step can be considered as the training phase. The training dataset is developed by collecting metrics such as Channel State Information (CSI) and RSS. This input feature is collected at each Reference Point (RP) from all Access Points (AP) [21]. Popular approaches for indoor positioning algorithms based on Wi-Fi fingerprinting include K-Nearest Neighbors (KNN) [5,22] as well as Generative Adversarial Network (GAN) variants such as Conditional GAN (CGAN) [23], Self-Normalizing GAN (SNGAN) [24], and Conditional Tabular GAN (CTGAN) [25]; other alternatives include Gaussian Process Regression (GPR) [26], Deep Neural Networks (DNN), and Random Forest (RF) [27]. All these are supervised learning based methods that require extensive data in order to be effectively trained. Generating a rich dataset every time is not practical, which brings forward the possibility of using unsupervised learning algorithms. Both phases can be made more effective by leveraging the temporal and spatial patterns of signals, using additional sensors, and relying on clever sensor fusion techniques such as Kalman filters [28]. Unlike fingerprinting methods, ranging methods can predict the final location of the user using trilateration or multilateration techniques [2,4].

To enhance positioning accuracy, the spatial relationships among APs can be effectively exploited by modeling their pairwise connectivity and optimizing AP placement. In [29,30], centimeter-level indoor positioning accuracy was achieved through precise AP localization. For graph-based modeling, inter-AP edges are typically weighted using the inverse of their physical distance, allowing the network to capture spatial proximity and signal correlations across APs. In parallel, temporal dependencies between consecutive WiFi scans can be modeled to mitigate the effects of noise and multipath fading. The study in [31] demonstrated these principles by integrating Graph Convolutional Networks (GCNs) and Temporal Convolutional Networks (TCNs) into a GTCN for processing CSI data. However, because CSI extraction requires specialized hardware and firmware modifications, such approaches have limited deployability on commodity WiFi devices.

Despite these approaches, several key limitations remain unaddressed. Existing spatial–temporal positioning frameworks rely almost exclusively on fine-grained CSI, which requires special hardware for implementation. Furthermore, graph-based positioning models typically employ a fixed AP topology derived from physical distances, which does not accurately reflect environment-dependent signal correlations shaped by multipath and NLOS propagation. Prior TCN-based architectures have been applied only to CSI measurements, and no work has exploited a TCN to jointly model WiFi RSS and RTT for indoor positioning. This work presents a novel approach utilizing a causal modeling framework that can jointly process RSS and RTT while adapting to AP connectivity. Inspired by this concept, we introduce a lightweight hybrid GTCN that jointly models spatial dependencies among APs through graph convolutions and the temporal dynamics of WiFi RSS and RTT measurements via causal dilated convolutions. By fusing both modalities, the proposed framework captures complementary spatial–temporal patterns while remaining hardware-compatible and easily deployable.

The proposed method is designed to operate in two stages: an offline phase and an online phase. During the offline phase, the model learns the spatial layout and signal characteristics of the environment using labeled WiFi scans collected at RPs. During the online phase, the system receives only real-time RSS and RTT measurements without requiring any labeled data and predicts the user’s position based on the learned spatial-temporal representation. This separation aligns with widely deployed fingerprinting-based positioning systems. To the best of our knowledge, this is the first study to employ a TCN-based architecture for WiFi RSS and RTT-based indoor positioning. The main contributions of this paper are summarized as follows:

- We propose an enhanced WiFi indoor positioning system that jointly utilizes RSS and RTT measurements. This hybrid approach exploits the complementary advantages of signal strength–based and time-of-flight–based ranging to achieve robust sub-meter accuracy across diverse environments, including LOS, NLOS, and mixed conditions.
- We introduce a computationally efficient GTCN that explicitly models spatial dependencies among APs using graph convolutions with inverse-distance edge weighting while concurrently capturing causal temporal correlations between consecutive signal scans through dilated TCNs. This unified design enhances both spatial consistency and temporal robustness while remaining suitable for real-time embedded deployment.
- We systematically investigate the impact of AP density under both LOS and NLOS conditions to assess the scalability and robustness of the proposed model. This analysis highlights the adaptability of the proposed hybrid GTCN model under both sparse and dense AP deployments.

The remainder of this paper is organized as follows: Section 2 reviews the related work, followed by the system model and the proposed methodology in Section 3; Section 4 presents the experimental datasets, implementation details, and performance evaluation results; finally, Section 5 concludes the paper and outlines potential directions for future research.

## 2. Related Work

The Fine Time Measurement (FTM) protocol in WiFi, introduced in the IEEE 802.11-2016 standard [32], can also offer high-precision distance estimates between a WiFi RTT-enabled smartphone and APs. This protocol measures the distance by sending ranging requests from the smartphone to the APs without needing to establish a connection, gathering both RSS and RTT data within a short time. Unlike the Time-of-Flight (ToF) method, FTM does not require clock synchronization between the smartphone and the APs, which makes the system simpler and more dependable. While fingerprinting using RSS and RTT measurements is popular, both have limitations specific to them. This includes high sensitivity to environmental conditions, limited spatial resolution, and more common NLOS effects. NLOS effects can be identified and removed, but if every valid signal is prone to NLOS conditions, removing them defeats the purpose. To mitigate these limitations, ref. [33] proposed combining RTT and RSS measurements while generating a fingerprinting map, resulting in improved position accuracy. In contrast to the conventional ToF methods that require clock synchronization between transmitter and receiver, the introduction of FTM enables high-precision ranging between smartphones and access points by measuring the RTT. This significantly reduces the time to obtain RTT and RSS readings within short sampling intervals, which makes this approach more practical and reliable [34]. Despite this development, RTT measurements and the resulting positioning estimates suffer greatly from NLOS conditions because of indoor obstacles and signal reflections. In recent years, WiFi RTT positioning has gained significant attention as a practical and standards-compliant approach for indoor positioning using IEEE 802.11mc and its successors. Early RTT-based works focused on improving ranging accuracy and mitigating multipath bias. A recent commercial-grade implementation, ref. [35] demonstrated the maturity of RTT-based indoor positioning using only commodity smartphones and access points. They employed a WiFi RTT positioning system using an Extended Kalman Filter (EKF) with random-walk and step-heading motion models, achieving sub-meter error across diverse walking datasets. Ref. [36] employed stack ensemble learning for indoor positioning, utilizing support vector regression and the XGBoost algorithm.

To improve robustness under mixed LOS and NLOS conditions, ref. [11] introduced an LOS identification and range calibration framework that combines Gaussian Process Regression (GPR)-based scenario recognition with LOS distance correction, yielding 0.99 m RMSE. In harsh multipath environments, ref. [37] extended this concept by employing GPR with Particle Swarm Optimization to model RTT measurement differences, reducing the mean error by 68.5% compared to least squares. There have been a number of other work works seeking to enhance the performance in complex NLOS conditions. Ref. [38] proposed an RTT localization algorithm with LOS compensation and trusted NLOS recognition using a Support Vector Machine (SVM) classifier and Bayesian selection, achieving a 53% improvement over baseline least-squares methods. A complementary work [39] presented an automated site survey approach that integrates GPS and smartphone sensors to determine AP coordinates for RTT-based localization, reducing pre-deployment effort while maintaining sub-meter accuracy.

Building on these RTT-based advancements for indoor positioning, researchers have increasingly explored hybrid RTT–RSS fusion methods that take advantage of the complementary properties of signal strength and time-of-flight measurements to improve positioning accuracy and robustness. The work in [40] introduced a hybrid WiFi RTT–RSS ranging framework that compensates for transmitter-side clock skew and calibrates RTT offsets, achieving a mean positioning error of 1.43 m with 0.19 s update intervals. Similarly, ref. [41] improved the classical RSS logarithmic path loss model using quadratic fitting and fuses calibrated RTT and RSS via adaptive weighting, yielding notable accuracy gains in complex indoor layouts. The study in [42] further analyzed the joint contribution of RTT and RSS features in fingerprinting, demonstrating that coupling both measurements can reduce network overhead while improving scalability and precision. From a learning perspective, ref. [43] proposed a Deep Fusion Model (RRLoc) that integrates time-based and fingerprinting features via deep canonical correlation analysis, improving localization accuracy by over 250% relative to conventional fingerprinting and multilateration methods. The dynamic model-switching approach proposed in [44] adaptively selects the optimal positioning model per environment using weighted machine learning, yielding up to 1.8 m improvement over traditional methods. To address multipath-induced instability, ref. [45] developed real-time NLOS/LOS identification algorithms based on short RSS/RTT sequences, achieving 96% discrimination accuracy with minimal latency.

Recent efforts have also explored multi-sensor fusion and optimization frameworks. Ref. [46] presented a tightly coupled integration platform that combines WiFi RTT, RSS, and MEMS-IMU data using adaptive filtering, achieving a 20% improvement in accuracy and robustness over standard EKF approaches. A similar concept was extended in [47] using Factor-Graph Optimization (FGO). Similarly, ref. [48] proposed implicit connectivity for GNN-based cooperative localization filter variants. In [49], a particle-filter-based system fused WiFi RTT, RSS, PDR, and map constraints using semi-parametric error models and collaborative optimization, resulting in meter-level accuracy within 0.19 s. These studies collectively underscore the potential of RTT and RSS fusion (often complemented by inertial or map-based constraints) to enhance robustness against multipath and NLOS effects. However, most existing methods process spatial and temporal information independently, without explicitly modeling the spatial graph topology of APs or the temporal evolution of signal dynamics. While CSI-based deep architectures can learn such spatiotemporal correlations, they require hardware modifications that limit deployment feasibility on commercial devices.

In [50], the authors introduced a mobility-induced graph learning framework that models user movement as graphs to enhance WiFi positioning accuracy, leveraging cross-graph and self-supervised learning to achieve robust performance without the need for labeled data. Ref. [51] applied graph neural networks to nonlinear regression in network localization, demonstrating their efficiency and robustness. Ref. [52] proposed a hybrid belief propagation–GNN framework that improves cooperative localization consistency without increasing complexity. Ref. [53] presented a vision-assisted privacy-preserving approach for generating data on large-scale WiFi positioning. Similarly, ref. [48] introduced implicit connectivity modeling and self-attention embeddings for cooperative localization. Refs. [54,55] demonstrated the strength of TCN for sequential modeling tasks, including Bluetooth-based indoor localization and human activity recognition. In parallel, recent studies have explored the application of TCN in WiFi-based sensing tasks such as human activity and interaction recognition. These works demonstrate the strong temporal modeling capabilities of TCNs in extracting discriminative motion patterns from wireless signals. For instance, ref. [56] proposed a Lightweight Mobile TCN (LM-TCN) that employs depthwise separable convolutions and gated residual mechanisms for low-power and memory-efficient human activity recognition across multiple indoor locations, achieving 95.2% accuracy while reducing computational cost to 6% of the baseline TCN. Ref. [57] introduced Wi-ATCN, an attentional TCN that integrates self-attention with WiFi CSI for human action prediction, improving performance stability across environments. More recently, [58] proposed WiFi-TCN, which combines temporal convolution, data augmentation, and attention mechanisms for efficient human–human interaction recognition, achieving 99.4% accuracy and demonstrating high generalization across subjects and scenarios.

While these TCN-based frameworks have demonstrated outstanding results for recognition tasks, they primarily focus on classifying human motion using high-resolution CSI data. In contrast, the proposed hybrid GTCN framework adapts the temporal modeling strength of TCNs to the problem of WiFi-based indoor positioning using low-dimensional RSS and RTT fingerprints. By jointly exploiting spatial graph reasoning among access points and causal temporal dependencies within signal sequences, our approach bridges the gap between lightweight fingerprinting and deep spatiotemporal learning, achieving robust and hardware-compatible sub-meter positioning performance.

## 3. System Model and Proposed Methodology

### 3.1. Preliminaries and Problem Formulation

This study addresses WiFi indoor positioning using measurements obtained directly from IEEE 802.11-compliant devices. We use WiFi RSS and RTT measurements as input features, both of which are measurable from commodity APs without requiring physical-layer modifications or dedicated ranging hardware. The objective is to estimate user coordinates in real time with a computationally efficient model suitable for embedded and edge platforms. All estimated position coordinates are expressed in a 2D Cartesian reference frame aligned with the floor layout, where each RP is mapped to its physical (x,y) position in meters.

Each environment is discretized into $N_{\mathrm{RP}}$ RPs. The coordinates of RP *r* are denoted by $y_{r}={\left[x_{r},y_{r}\right]}^{⊤}$ in meters, determined by a grid scale factor *g* derived from the floor layout. At each RP, WiFi scans are collected from up to *N* APs. The *t*-th measurement from AP *i* at RP *r* is

$$\left(x_{r,t,i}^{\mathrm{rss}},\;x_{r,t,i}^{\mathrm{rtt}}\right),$$

where $x_{r,t,i}^{\mathrm{rss}}$ is the RSS (dBm) and $x_{r,t,i}^{\mathrm{rtt}}$ is the RTT (mm).

For each RP *r*, the RSS and RTT samples form

$$R_{r}^{\left(\mathrm{rss}\right)}\in R^{T_{r}\times N},\;R_{r}^{\left(\mathrm{rtt}\right)}\in R^{T_{r}\times N},$$

where $T_{r}$ is the number of scans at RP *r*. The complete fingerprint database is

$$D={\left\{R_{r}^{\left(\mathrm{rss}\right)},\;R_{r}^{\left(\mathrm{rtt}\right)},\;y_{r}\right\}}_{r=1}^{N_{\mathrm{RP}}}.$$

The database is segmented into temporal windows of fixed length *T* to construct training sequences $X\in R^{T\times N\times F}$, where *F* is the feature dimension. Each sequence corresponds to one RP label $y_{r}$.

#### Signal Modeling and Objective

WiFi RSS and RTT measurements provide complementary information on the propagation channel. RSS reflects large-scale attenuation and shadowing, while RTT captures geometric propagation delay. Both are affected by multipath, hardware bias, and NLOS conditions; however, their error characteristics are only partially correlated. Integrating WiFi RTT and RSS measurements enables the model to mitigate individual weaknesses such as multipath distortion in RTT and shadowing in RSS, resulting in improved overall positioning accuracy.

For each AP *i* and time *t*, we construct a composite feature vector

(1) $$f_{t,i}=\left[x_{t,i}^{\mathrm{rss}},\;x_{t,i}^{\mathrm{rtt}},\;m_{t,i}^{\mathrm{rss}},\;m_{t,i}^{\mathrm{rtt}},\;\phi_{t,i}^{\mathrm{stat}},\;{\hat{x}}_{i},\;{\hat{y}}_{i}\right],$$

where $m_{t,i}^{\mathrm{rss}}$ and $m_{t,i}^{\mathrm{rtt}}$ are binary validity masks indicating whether the RSS and RTT measurements from AP *i* at time *t* are valid or missing/invalid. These masks prevent placeholder values (e.g., extremely low RSS or saturated RTT) from biasing training and allow the network to down-weight unreliable observations. Here, $\phi_{t,i}^{\mathrm{stat}}$ denotes rolling statistical descriptors (mean, median, standard deviation, skewness, and kurtosis) computed over a causal sliding window of past RSS/RTT samples, while $\left({\hat{x}}_{i},{\hat{y}}_{i}\right)$ represent normalized AP coordinates that are set to zero when unavailable.

Stacking features across APs yields

(2) $$F_{t}=\left[\begin{array}{c} f_{t,1}^{⊤}\\ \vdots \\ f_{t,N}^{⊤} \end{array}\right]\in R^{N\times F}$$

and a spatiotemporal input tensor over *T* scans:

(3) $$X_{1:T}=\left\{F_{1},\ldots ,F_{T}\right\}\in R^{T\times N\times F}.$$

Given the causal input $X_{1:t}$, the model $f_{\theta }$ predicts

(4) $${\hat{y}}_{t}=f_{\theta }\left(X_{1:t}\right)={\left[{\hat{x}}_{t},{\hat{y}}_{t}\right]}^{⊤},$$

where ${\hat{y}}_{t}$ depends only on observations up to time *t*.

The parameters θ are optimized by minimizing the total positioning loss

(5) $$L\left(\theta \right)=\frac{1}{N_{s}}\sum_{i=1}^{N_{s}}\left[\phi_{\beta }\;\left({\hat{y}}_{i}^{\left(n\right)}-y_{i}^{\left(n\right)}\right)+w_{E}{∥{\hat{y}}_{i}-y_{i}∥}_{2}\right]+R\left(\theta \right),$$

where $N_{s}$ is the total number of training samples (temporal windows), $\phi_{\beta }\left(\cdot \right)$ denotes the Huber loss with threshold β, $w_{E}$ weights the Euclidean positioning error, ${\left(\cdot \right)}^{\left(n\right)}$ represents normalized coordinates, and R(θ) aggregates regularization terms for the adaptive graph and node gating.

The Huber loss [59] is defined as

(6) $$\phi_{\beta }\left(a\right)=\left\{\begin{array}{cc} \frac{1}{2}a^{2}, & \mathrm{if}\;|a|\leq \beta ,\\ \beta (|a|-\frac{1}{2}\beta ), & \mathrm{otherwise}, \end{array}\right.$$

where $a=y-\hat{y}$ denotes the residual error and β is the transition parameter between the quadratic and linear regions. This formulation behaves like an $\ell_{2}$ loss for small residuals and an $\ell_{1}$ loss for large residuals, providing robustness against outliers in RSS and RTT measurements.

The optimal network parameters are obtained as

(7) $$\theta^{*}=\arg\min_{\theta }L\left(\theta \right),$$

subject to the causality constraint in (4). This formulation enables joint learning of spatial correlations among APs, temporal consistency in RSS/RTT measurements, and a stable mapping from features to user position. The learned mapping $f_{\theta }\left(\cdot \right)$ forms the basis of the proposed GTCN, which is described next.

### 3.2. Proposed Indoor Positioning Method

**Figure 1:**
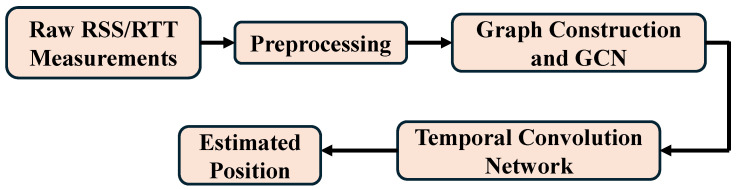
High-level overview of the proposed WiFi indoor positioning architecture.

Inspired by recent GTCN-based CSI localization frameworks [31], we extend the graph temporal paradigm to WiFi RSS and RTT fingerprinting using only commodity WiFi measurements. The proposed model consists of (i) a GCN to capture spatial correlations among APs and (ii) a TCN with causal dilated convolutions to capture temporal dynamics. Their integration yields a unified, causal, and computationally efficient spatiotemporal architecture. A high-level overview of the proposed pipeline is illustrated in Figure 1, showing that raw RSS and RTT measurements are first preprocessed, followed by graph construction, GCN-based spatial modeling, TCN-based temporal modeling, and estimating the position.

#### 3.2.1. GCN: Spatial Correlation Modeling

We represent the AP layout as a weighted undirected graph G=(V,E), where each node corresponds to an AP and edges encode spatial proximity. The adjacency matrix A is defined as

(8) $$A_{ij}=A_{ji}=\left\{\begin{array}{cc} 0, & i=j,\\ \frac{1}{d_{ij}}, & i\neq j, \end{array}\right.$$

where $d_{ij}$ is the Euclidean distance between the *i*-th and *j*-th APs. This construction emphasizes closer AP pairs while suppressing weak long-range couplings. We denote the adjacency in (8) as the base matrix $A_{\mathrm{base}}$, representing the geometry-driven AP connectivity used as the structural prior for later adaptive refinement.

To ensure numerical stability and include self-connections, we define

(9) $$\tilde{A}=A+I,\;\tilde{D}=\mathrm{diag}\;\left(\sum_{j}{\tilde{A}}_{ij}\right)$$

and use the symmetrically normalized adjacency ${\tilde{D}}^{-\frac{1}{2}}\tilde{A}{\tilde{D}}^{-\frac{1}{2}}$. Given an input feature matrix $X\in R^{N\times F_{\mathrm{in}}}$ at a single time step (e.g., one row of $F_{t}$ in (3)), a GCN layer produces

(10) $$Y=\sigma \;\left({\tilde{D}}^{-\frac{1}{2}}\tilde{A}{\tilde{D}}^{-\frac{1}{2}}XW\right),$$

where $W\in R^{F_{\mathrm{in}}\times F_{\mathrm{out}}}$ is a learnable weight matrix and σ(·) is a nonlinear activation (ReLU). Stacking multiple GCN layers allows each AP to aggregate information from multi-hop neighbors, yielding spatially smoothed and topology-aware representations.

In the proposed framework, the GCN is applied to per-scan features across APs, producing spatially enriched node embeddings that serve as input to the temporal modeling stage.

While the geometry-based adjacency in (8) encodes spatial proximity, it cannot capture environment-dependent signal relationships such as multipath clusters, shared NLOS effects, or correlated RSS and RTT fluctuations. To address this, the model incorporates a learnable residual adjacency that refines inter-AP connectivity in a data-driven manner. A symmetric bounded residual component is defined as

(11) $$R=\tanh\;\left(\frac{1}{2}\left(S+S^{⊤}\right)\right),$$

where $S\in R^{N\times N}$ is the trainable matrix and the effective adjacency becomes

(12) $$A_{\mathrm{eff}}=A_{\mathrm{base}}+\alpha \;R,\;\alpha \in \left[0,1\right],$$

where α is a learnable mixing weight. We then form the normalized adaptive adjacency

(13) $${\tilde{A}}_{\mathrm{eff}}=A_{\mathrm{eff}}+I,\;{\tilde{D}}_{\mathrm{eff}}=\mathrm{diag}\;\left(\sum_{j}{\tilde{A}}_{\mathrm{eff},ij}\right),\;\hat{A}={\tilde{D}}_{\mathrm{eff}}^{-\frac{1}{2}}{\tilde{A}}_{\mathrm{eff}}{\tilde{D}}_{\mathrm{eff}}^{-\frac{1}{2}}.$$

**Figure 2:**
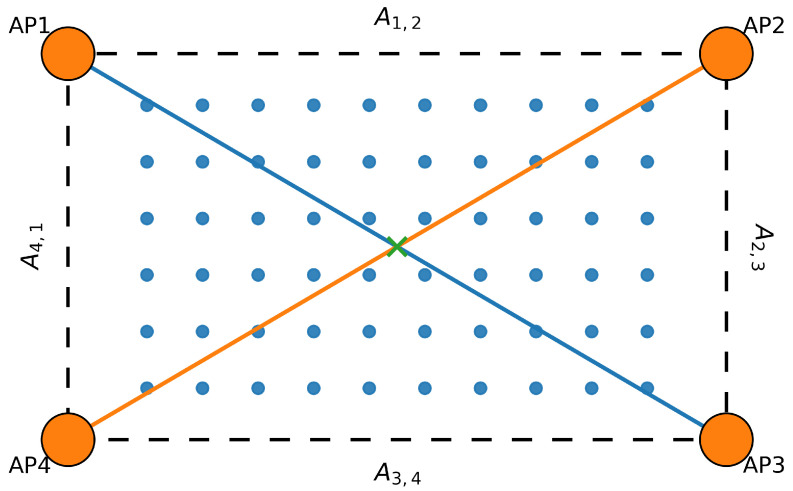
Graph representation of the AP topology. Nodes correspond to APs, while edge weights are defined as the inverse of the Euclidean distance between APs. This spatial graph underlies the GCN-based feature propagation in (10).

Here, $\hat{A}$ replaces the normalized adjacency in (10), enabling the GCN to leverage both structural geometry and data-driven AP correlations. The model further employs a node gating mechanism that modulates each AP’s contribution based on the reliability of its RSS and RTT features. For the feature vector $f_{t,i}$ of AP *i* at time *t*, a gating weight is computed as

(14) $$w_{t,i}=\sigma \;\left(\frac{g(f_{t,i})}{\tau }\right),\;w_{t,i}\in \left(0,1\right],$$

where g(·) is a small MLP, σ(·) is the sigmoid function, and τ>0 is a learnable temperature controlling gating sharpness. The gated node features are then

(15) $${\tilde{f}}_{t,i}=w_{t,i}\;f_{t,i},$$

allowing reliable APs to be emphasized while noisy or unstable APs are down-weighted. As illustrated in Figure 2, the four APs form a weighted graph where, for instance, $A_{1,2}=1/d_{1,2}$ and $A_{2,3}=1/d_{2,3}$. Physically closer APs yield larger edge weights, reinforcing local spatial interactions.

#### 3.2.2. TCN: Causal Sequence Modeling

To capture the temporal evolution of WiFi RSS and RTT fingerprints, we employ a TCN with dilated causal convolutions [60]. This design ensures that the output at time *t* depends only on ${\left\{x_{\tau }\right\}}_{\tau \leq t}$, which is essential for real-time tracking.

For an input feature sequence $X={\left[x_{1},x_{2},\ldots ,x_{T}\right]}^{⊤}$, a dilated causal convolutional layer with kernel size *k* and dilation factor *d* computes the output sequence $Y={\left[y_{1},\ldots ,y_{T}\right]}^{⊤}$ as

(16) $$y_{t}=\sum_{i=0}^{k-1}W_{i}\;x_{t-d\cdot i}+b,$$

where $W_{i}$ are learnable filters, b is a bias term, and indices with t−d·i<1 are handled via causal padding. Equivalently, this can be written compactly as

(17) $$Y=f\;\left(W{*}_{d}X+b\right),$$

where ${*}_{d}$ denotes the dilated causal convolution.

**Figure 3:**
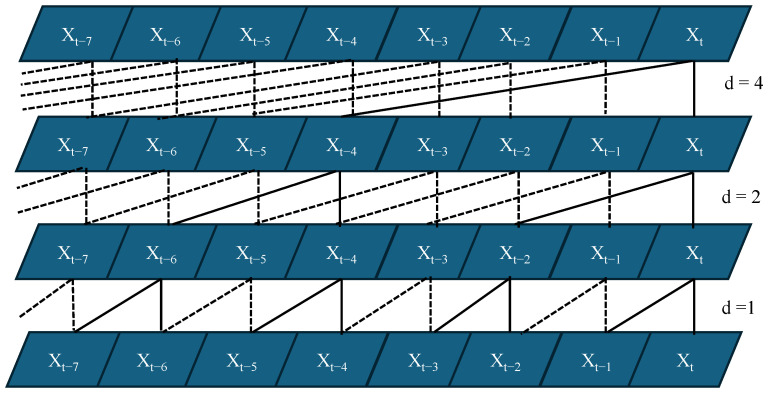
A TCN layer structure.

**Figure 4:**
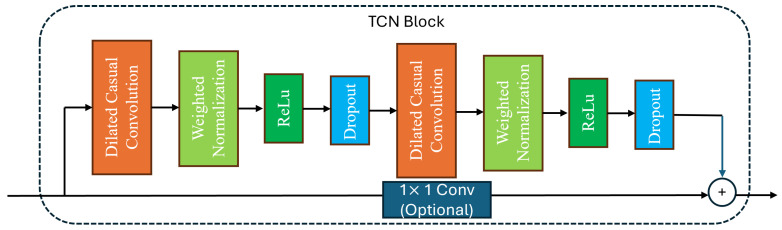
TCN detail block diagram.

Residual connections are incorporated to stabilize training and mitigate gradient degradation:

(18) $$O=\sigma \;\left(X+F(X)\right),$$

where F(X) denotes the sequence of operations inside a residual block (two dilated causal convolutions with normalization, activation, and dropout), and a 1×1 convolution is used on the skip path when channel dimensions differ. By stacking residual blocks with dilation factors d∈{1,2,4}, the receptive field grows exponentially, enabling the TCN to capture both short-term variations and long-range dependencies in RSS and RTT sequences without violating causality, as shown in Figure 3 and Figure 4.

#### 3.2.3. Hybrid GTCN Model

In the proposed hybrid GTCN model, the GCN extracts spatial relationships among APs, while the TCN captures temporal dependencies in the sequence of WiFi RSS and RTT fingerprints. Together, they form the hybrid GTCN, which jointly learns spatial–temporal correlations for robust indoor positioning. At each time step, preprocessed WiFi RSS and RTT fingerprints are passed through stacked GCN layers that operate on the AP adjacency graph, as shown in Figure 2. These layers aggregate neighborhood information and generate topology-aware node embeddings. A weighted node pooling module then fuses the node features into a compact global representation of the wireless environment for that scan. The sequence of pooled embeddings across consecutive time windows is fed into a stack of dilated causal convolutions (the TCN block) to model temporal continuity and motion patterns, as shown in Figure 3. The final regression head maps the temporal feature vector to a 2D position estimate ${\hat{y}}_{t}={\left[{\hat{x}}_{t},{\hat{y}}_{t}\right]}^{⊤}$.

**Figure 5:**
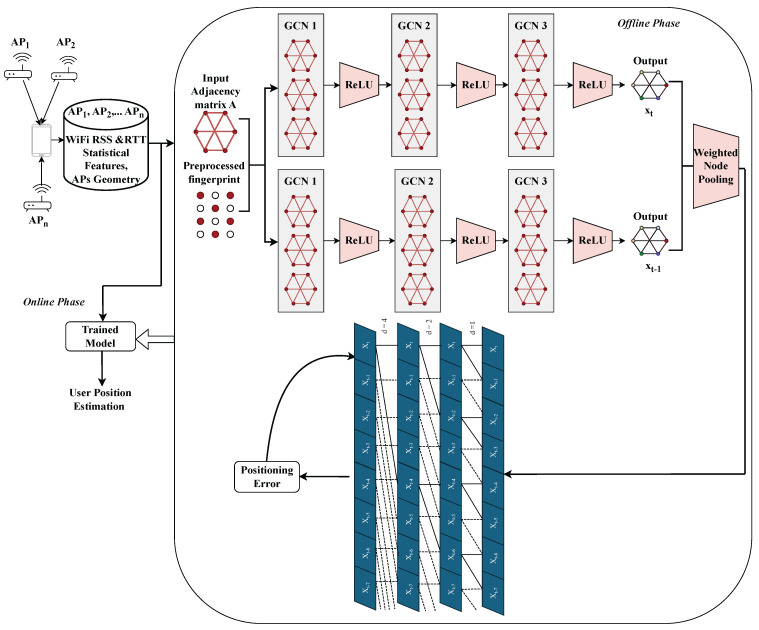
Overall architecture of the proposed hybrid GTCN model for WiFi indoor positioning using RSS and RTT features. The GCN layers capture spatial dependencies among APs via the adjacency matrix, while the TCN layers exploit temporal dynamics through dilated causal convolutions. Weighted node pooling and residual connections ensure efficient feature fusion, leading to robust position estimation under multipath and NLOS conditions.

The overall end-to-end process is illustrated in Figure 5, and the algorithm is described in Algorithm 1. The pipeline begins with data collection from multiple APs, extraction of statistical WiFi RSS and RTT features, and formation of the spatial graph using AP geometry. These data feed the GCN layers (for spatial embedding) and the TCN layers (for temporal modeling) during offline phase. The model outputs the estimated position, which is compared against the ground-truth coordinates to compute the positioning error during online phase. The network parameters are updated according to the loss function in (5), combining Huber and Euclidean terms.
**Algorithm 1:** Proposed Hybrid GTCN-Based WiFi Indoor Positioning Method   **Input**: Raw WiFi RSS and RTT fingerprint sequences; optional AP coordinates   **Output**: Estimated positions $\hat{y}={\left[\hat{x},\hat{y}\right]}^{⊤}$_**1**_ **Initialization:** Initialize model parameters._**2**_ **Offline Phase:**   **Step 1. Preprocessing:**Load raw WiFi RSS and RTT scans for each environment.Compute per-AP statistics and normalize WiFi RSS and RTT measurements.Augment inputs with causal rolling-window statistics (mean, median, std, skewness, kurtosis).Encode AP coordinates (z-scored (x,y)) as node features if available.Group scans by RP into sequences of length *T*.   **Step 2. Graph Construction:**Build base adjacency $A_{\mathrm{base}}$ from inverse inter-AP distances.Learn symmetric residual adjacency R and a mixing weight α.Form normalized adaptive graph:$$\hat{A}=D^{-1/2}\left(A_{\mathrm{base}}+\alpha R+I\right)D^{-1/2}.$$   **Step 3. Spatial Encoding (GCN + Node Gating):**For each time step and AP, compute a gating weight using a lightweight MLP.Apply gating to node features and propagate them through GCN layers using $\hat{A}$.Gating adaptively emphasizes reliable APs and down-weights noisy or unstable ones.   **Step 4. Temporal Modeling (Causal TCN):**Aggregate gated per-AP embeddings at each time step to obtain a compact temporal sequence.Feed the sequence into stacked dilated causal TCN blocks to capture temporal dependencies without future leakage.   **Step 5. Localization and Optimization:**Map the final TCN output to normalized coordinates ${\hat{y}}^{\left(n\right)}={\left[{\hat{x}}^{\left(n\right)},{\hat{y}}^{\left(n\right)}\right]}^{⊤}$.Compute Huber loss on normalized coordinates and Euclidean error on denormalized predictions.Update all parameters using AdamW with gradient clipping.   **Online Phase:**Apply the same preprocessing (using training statistics) to incoming RSS/RTT scans.Form sequences of length *T* and pass them through the trained GTCN.Output the estimated position $\hat{y}={\left[\hat{x},\hat{y}\right]}^{⊤}$.

## 4. Experimental Dataset and Results

### 4.1. Data Collection and Testbeds

The proposed GTCN model is evaluated using the publicly available WiFi RSS/RTT dataset introduced by [44]. The dataset contains measurements from four representative indoor environments: Lecture Theatre, Office, Corridor, and Building Floor, each exhibiting different LOS and NLOS characteristics. All measurements were collected with an LG G8X ThinQ smartphone equipped with IEEE 802.11mc-compliant hardware and a set of commercial APs. For each RP, RSS (dBm) and RTT (mm) were recorded simultaneously from all visible APs. The dataset assumes a standard WiFi RTT measurement setup in which the smartphone acts as the initiating station and the APs operate as responders. Each testbed is discretized using a uniform 0.6×0.6 m grid, and the corresponding ground-truth coordinates are provided in the dataset. At every RP, multiple WiFi scans were performed to capture temporal variations: 60 scans per RP in the Lecture Theatre, Office, and Corridor and 120 scans per RP in the larger Building Floor environment. Data collection included both LOS and obstructed NLOS links, producing a diverse set of propagation conditions suitable for benchmarking spatiotemporal learning models.

**Figure 6:**
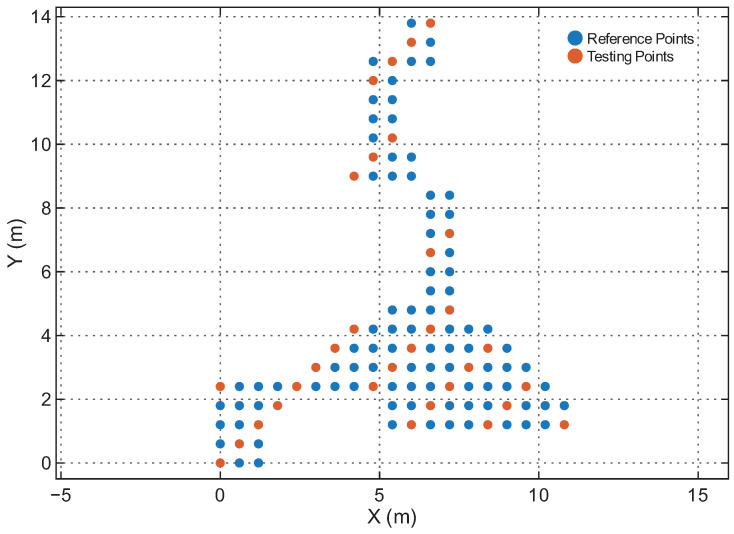
Reference and testing points in the Lecture Theatre.

**Figure 7:**
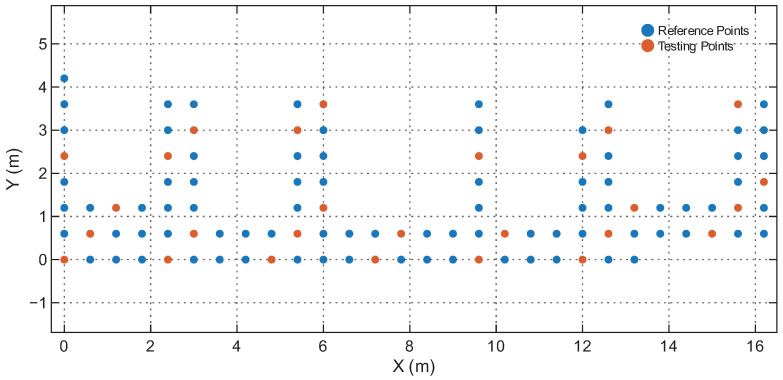
Reference and testing points in the Office.

**Figure 8:**
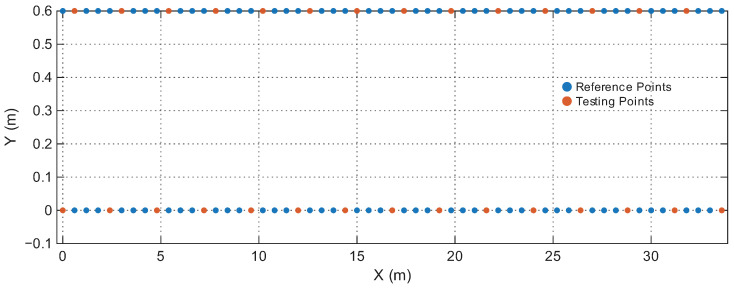
Reference and testing points in the Corridor.

**Figure 9:**
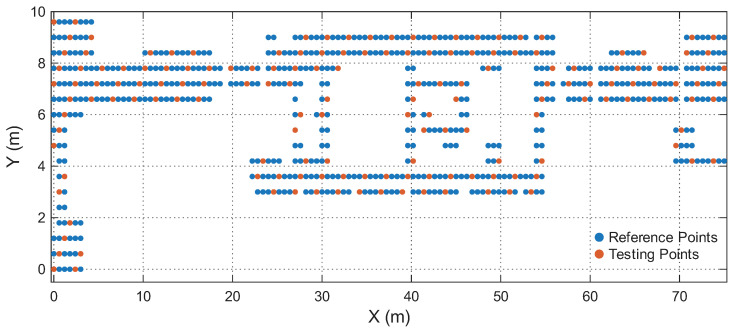
Reference and testing points in the Building floor.

Table 1 summarizes the four environments. The RPs and Testing Points (TPs) are spatially disjoint in all cases, ensuring that the model is evaluated on unseen locations rather than memorized grid points. Figure 6, Figure 7, Figure 8 and Figure 9 illustrate the spatial layouts and the disjoint training/testing partitions for all environments. The four environments were selected to capture a representative range of indoor conditions: compact LOS spaces, mixed-LOS office layouts, elongated NLOS corridors, and a large heterogeneous floor plan, enabling systematic evaluation of model generalization. To ensure fair comparison with prior studies, we adopt the same training–testing splits provided in [44]. The positioning performance is evaluated using the Root Mean Square Error (RMSE) of the 2D position estimates and the empirical Cumulative Distribution Function (CDF) of the localization error. Let $y_{i}={\left[x_{i},y_{i}\right]}^{⊤}$ and ${\hat{y}}_{i}={\left[{\hat{x}}_{i},{\hat{y}}_{i}\right]}^{⊤}$ denote the true and estimated coordinates of the *i*-th test sample, respectively. The instantaneous Euclidean localization error is provided by

(19) $$d_{i}={∥y_{i}-{\hat{y}}_{i}∥}_{2}.$$

The overall positioning accuracy is evaluated using the RMSE metric:

(20) $$\mathrm{RMSE}=\sqrt{\frac{1}{N}\sum_{i=1}^{N}\left[{\left(x_{i}-{\hat{x}}_{i}\right)}^{2}+{\left(y_{i}-{\hat{y}}_{i}\right)}^{2}\right]},$$

where *N* is the total number of test samples. A lower RMSE indicates higher average positioning accuracy. To provide a statistical view of localization performance, the empirical CDF is also reported. The CDF curve illustrates the probability that the positioning error does not exceed a given threshold, offering an interpretable measure of the model’s reliability across test environments.

### 4.2. Model Parameters

The proposed GTCN model was implemented with the hyperparameter configurations summarized in Table 2. The architecture comprises three GCN layers for spatial feature extraction and three TCN blocks with causal dilated convolutions for temporal modeling. This combination efficiently captures both inter-AP spatial relationships and time-varying signal dynamics while maintaining computational efficiency suitable for embedded deployment. A hidden feature dimension of 64 was selected as a balanced tradeoff between model expressiveness and complexity, yielding stable training behavior and avoiding overfitting on smaller datasets. The causal TCN blocks use dilation rates (1,2,4) to exponentially expand the receptive field without increasing kernel size or depth. The model was trained using the AdamW optimizer with a learning rate of $1\times 10^{-3}$ and a weight decay of $1\times 10^{-4}$ to stabilize convergence and mitigate overfitting. Huber and Euclidean losses are combined with weights $\left(w_{H},w_{E}\right)=\left(1.0,0.5\right)$ to ensure robustness to outliers in RSS and RTT measurements while maintaining smooth gradient behavior.

**Figure 10:**
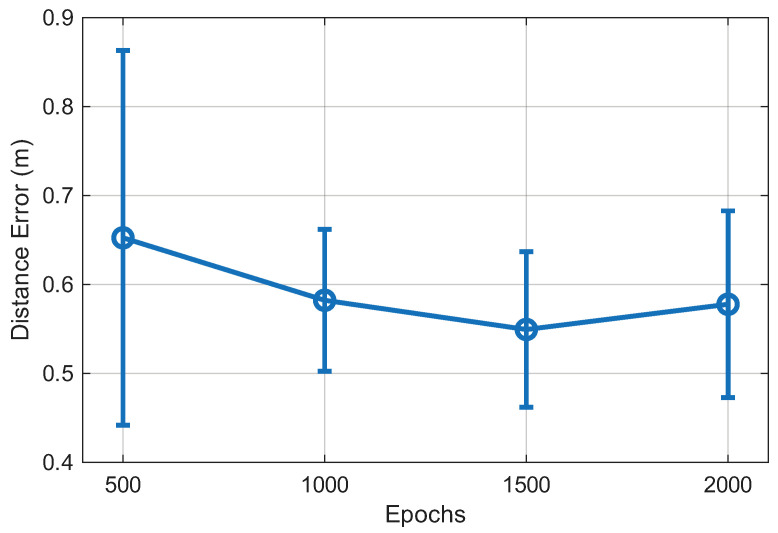
Effect of training epochs on the positioning error of the proposed GTCN model.

Figure 10 illustrates the effect of the training epochs on the positioning accuracy of the proposed GTCN model. The average distance error gradually decreases as the number of epochs increases, reflecting steady optimization of network parameters. After approximately 1000 epochs, the model converges with a stable distance error of around 0.55 m, indicating that the proposed framework effectively learns spatial–temporal dependencies without overfitting. This convergence behavior demonstrates the reliability and robustness of the GTCN model across different indoor environments. Although a slight increase in the mean error is observed at 2000 epochs, this variation remains within the error-bar range. The model effectively stabilizes between 1000 and 1500 epochs. Therefore, we selected 1500 epochs as the default training setting for all experiments. This choice provides a balanced tradeoff between training time and stable convergence while avoiding unnecessary computation beyond the point of meaningful improvement.

### 4.3. Positioning Performance

#### 4.3.1. Comparison of RSS, RTT, and Hybrid RSS–RTT

We first evaluate the contributions of different input modalities: RSS-only, RTT-only, and combined RSS+RTT fingerprints, which is denoted as “Both”. Table 3 reports the positioning RMSE across four environments and compares the proposed method with conventional fingerprinting and learning-based approaches such as MT-SDAE [61], RS-stacking [62], NWEC [63], and the Dynamic Model [44]. Across all testbeds, RTT-only consistently outperforms RSS-only, confirming the advantage of geometric time-of-flight information under both LOS and NLOS conditions. The proposed model with both outperforms all existing baseline methods across the Office, Corridor, and Building environments, achieving RMSE values of 0.560 m, 0.529 m, and 0.660 m surpassing the Dynamic Model with RMSE 0.698 m, 0.569 m, and 0.950 m, respectively. RTT-only also shows strong performance in these testbeds, reducing RMSE to 0.649 m in the Office and 0.731 m in the Building, compared to much higher RSS-only errors of 1.203 m and 1.579 m. In the Lecture Theatre, RSS-only again yields the largest error 2.170 m, while RTT-only achieves the lowest RMSE of 0.449 m. Because RTT is already highly reliable in this open LOS-dominant environment, the hybrid configuration with RMSE 0.501 m does not surpass RTT-only. A similar trend was also reported in the Dynamic Model work [44], where RTT outperformed RSS in the same testbed.

**Figure 11:**
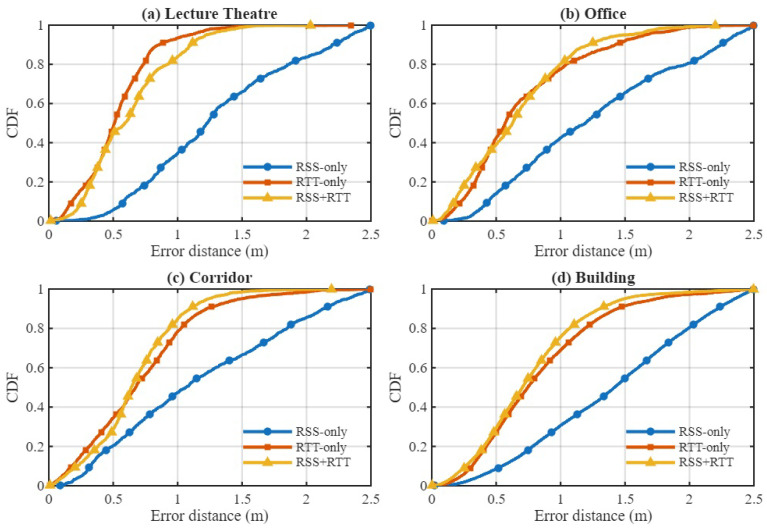
CDF of positioning error for RSS-only, RTT-only, and hybrid RSS+RTT across four environments using the proposed hybrid GTCN model.

Figure 11 shows the CDFs of the positioning error for RSS-only, RTT-only, and hybrid RSS–RTT per environment. The CDFs align with the RMSE trends: RSS-only exhibits the highest error spread due to sensitivity to multipath and device orientation, while RTT-only provides tighter error curves and improved stability. The hybrid RSS–RTT mode generally achieves the best accuracy, especially in heterogeneous areas such as the Office, Corridor, and Building environments. In contrast, in the Lecture Theatre, where propagation is dominated by strong LOS paths (as shown in Figure 6), the additional RSS information provides limited benefit, explaining why the hybrid mode does not outperform RTT-only. The hybrid configuration achieves sub-meter accuracy in the Office, Corridor, and Building environments and remains competitive in the Lecture Theatre, demonstrating the model’s ability to effectively exploit complementary RSS and RTT features and capture the underlying spatiotemporal structure of WiFi fingerprints.

To ensure a fair comparison with prior work, we also report the average single CPU time required to process a single WiFi scan, following the protocol used in [61,62,63]. The learning-based baselines JMT-SDAE, RS-stacking, and NWEC require approximately 0.0297 ms, 0.0466 ms, and 0.0250 ms per scan, respectively, while the Dynamic Model [44] requires 0.0118 ms per scan. The proposed model operates at 0.0211 ms per scan, which is slightly higher than the Dynamic Model yet remains well within the sub-millisecond latency range. Importantly, our model reduces the RMSE from 0.95 m to 0.66 m in the Building, which is the largest and most complex environment with mixed LOS and NLOS paths, offering a favorable accuracy–latency tradeoff while retaining real-time suitability for edge devices. We also report the training cost of the proposed model on the Building dataset, which required approximately 38 min to train for 1500 epochs. Because training is performed offline and only inference runs on the edge, this one-time cost does not impact real-time operation. Combined with its low inference latency, the proposed model remains lightweight and practical, delivering lower positioning RMSE.

#### 4.3.2. Comparison of GCN, TCN, and Hybrid GTCN Model

**Figure 12:**
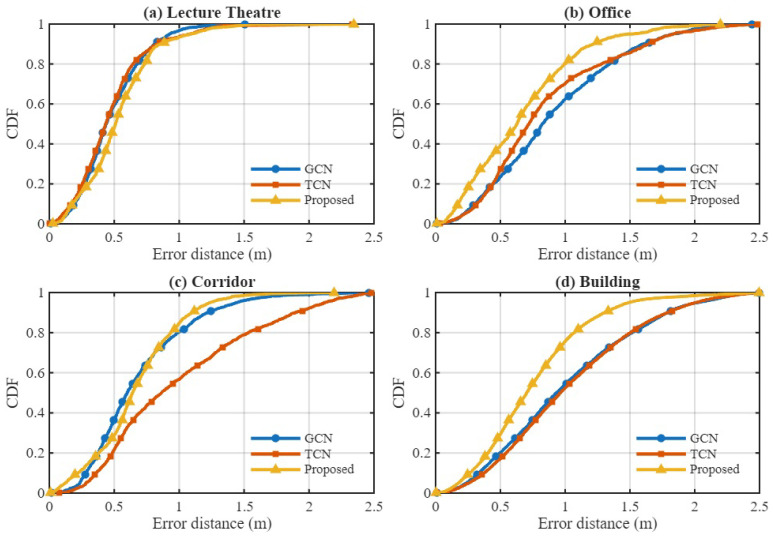
Per-environment CDF comparison of GCN, TCN, and the proposed hybrid GTCN model using RSS and RTT features.

Next, we compare three model architectures trained under identical conditions: a GCN, TCN, and the proposed GTCN. Figure 12 depicts the environment-wise CDFs of the positioning error. For consistency, all models in the Lecture Theatre were evaluated using their best RTT-based configurations. The hybrid GTCN model consistently outperforms the standalone GCN and TCN across most test environments. In compact and predominantly LOS conditions such as the Lecture Theatre and Office, the GTCN exhibits a steeper CDF curve in the low-error region, reflecting enhanced sub-meter reliability and faster convergence of positioning accuracy. In the Corridor, where propagation is largely NLOS with strong multipath reflections, the GTCN surpasses the TCN by effectively exploiting graph-based spatial context among APs, confirming the benefit of incorporating AP topology into temporal modeling.

The Building Floor testbed, which is the largest and most heterogeneous environment (92 m × 15 m, mixed LOS–NLOS), poses the most significant challenge due to its rich structural complexity and varying AP visibility. Here, the proposed GTCN model yields the highest probability of errors below 1 m, demonstrating strong scalability and generalization in large-scale deployments. Overall, the results show that RTT-based features dominate in pure LOS conditions (the Lecture Theatre) because of their direct geometric range correlation, while the combined RSS and RTT features within the GTCN offers superior robustness in mixed and NLOS environments (Office, Corridor, and Building). These findings confirm that jointly modeling spatial AP relationships and causal temporal dynamics is crucial for robust fingerprinting-based indoor positioning under diverse propagation conditions. In addition to the CDF-based comparison, Table 4 summarizes the RMSE performance of all three model variants across the four environments for different feature modes. The results further reinforce the advantages of the proposed hybrid GTCN model in terms of positioning performance. In the Lecture Theatre, the TCN achieves the lowest RMSE, consistent with the earlier observation that RTT dominates in clean LOS settings. However, in the Office, Corridor, and Building environments, which all exhibit stronger NLOS behavior, richer multipath, and more irregular AP visibility, the proposed GTCN achieves the lowest RMSE among all models. This performance gain highlights the benefit of combining spatial graph reasoning via the GCN with temporal sequence modeling via the TCN.

**Figure 13:**
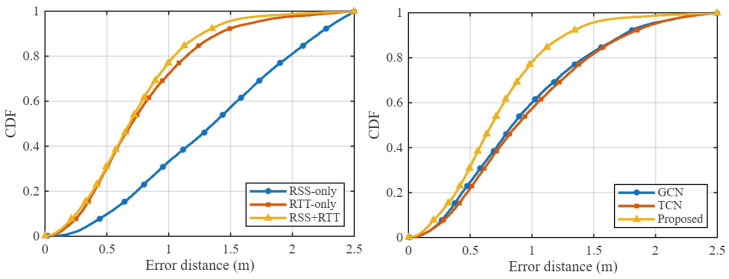
Aggregated CDF of positioning error based on input features and models across all four environments.

Specifically, the proposed GTCN reduces the RMSE by 10.0% in the Office, 9.7% in the Corridor, and 12.8% in the Building compared to the best-performing baseline in each environment. These improvements align with the aggregated CDFs shown in Figure 13, showing that the proposed GTCN model consistently produces smaller errors and a higher fraction of sub-meter predictions under challenging propagation conditions. These results provide quantitative confirmation that the proposed GTCN offers a balanced and scalable solution, performing competitively in LOS scenarios and delivering superior robustness in NLOS and large-scale environments.

#### 4.3.3. Impact of Number of APs

We further analyze the sensitivity of the proposed framework to the number of deployed APs. For each environment, the best-performing configuration (feature mode and model) is evaluated as the AP count varies, starting from a minimum of three. This lower bound was chosen because at least three APs are required to perform 2D indoor positioning via geometric trilateration, ensuring sufficient spatial diversity for reliable localization.

**Figure 14:**
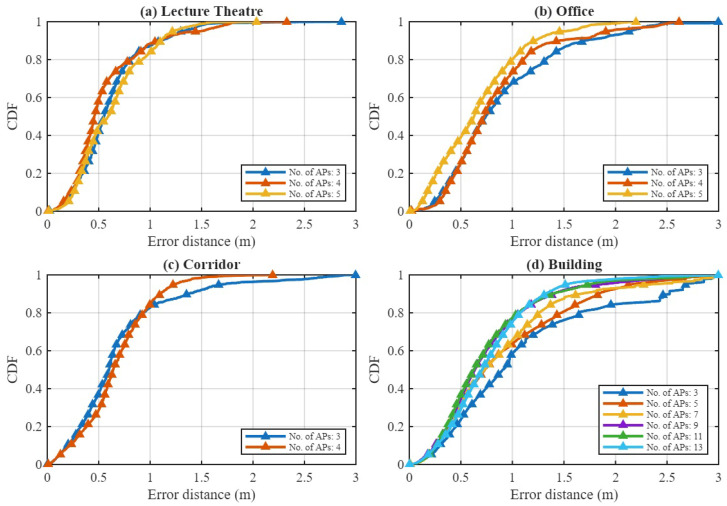
CDF of positioning error for varying numbers of APs across the four environments.

Table 5, Table 6, Table 7 and Table 8 summarize the positioning RMSE, while Figure 14 presents the corresponding CDFs of the positioning error. The results demonstrate that increasing AP density systematically enhances positioning accuracy, although the degree of improvement depends on the environmental characteristics. In the Lecture Theatre (compact LOS environment), the performance saturates beyond four APs, suggesting that a moderate deployment already provides sufficient geometric diversity for accurate time-of-flight estimation. Similarly, in the Office (mixed LOS–NLOS scenario), adding up to five APs significantly improves robustness, with the combination of RSS and RTT feature mode achieving 0.56 m RMSE and stable sub-meter performance thereafter.

In the Corridor (elongated NLOS environment), where multipath and reflection effects are dominant, the addition of even a single AP noticeably improves the probability of sub-meter accuracy by providing redundant spatial information to the GCN component. The most pronounced effect is observed in the Building Floor (large-scale mixed LOS–NLOS layout). As the number of APs increases from 3 to 13, the RTT-only RMSE decreases from over 9 m to below 1 m, while the combination of RSS and RTT configuration achieves 0.66 m RMSE. This strong monotonic gain highlights the scalability of the proposed framework in large and structurally complex indoor environments.

Overall, the proposed hybrid GTCN model effectively exploits additional APs when available while maintaining robust performance under sparse deployments. These findings indicate that while dense AP coverage benefits large, heterogeneous spaces, compact environments can achieve comparable accuracy with only a few strategically placed APs when leveraging the joint spatial–temporal learning capability of the proposed model.

#### 4.3.4. Model Complexity

We assess the computational complexity of the GCN, TCN, and hybrid GTCN models under their best-performing configurations in Table 9, which reports the total number of trainable parameters and Floating-Point Operations (FLOPs) in the causal setting. Trainable parameters represent the total number of learnable weights and biases within a model. This value indicates the storage and memory footprint during both training and inference, directly reflecting the model size. Floating-Point Operations (FLOPs) quantify the total arithmetical operations (multiplications and additions) required to process one input sequence, providing an estimate of the computational workload and inference latency.

Among the compared architectures, the GCN exhibits the lowest complexity and is suitable for highly resource-constrained devices, although it lacks temporal modeling. The TCN effectively captures temporal dependencies but does not encode explicit AP topology. The proposed hybrid GTCN introduces only a modest computational overhead relative to TCN while consistently achieving the best positioning accuracy. With fewer than $10^{5}$ trainable parameters and tens of MFLOPs per sequence, the GTCN offers an optimal balance between accuracy and efficiency, supporting real-time deployment on embedded and edge devices.

## 5. Conclusions

This study proposes a lightweight GTCN for hybrid WiFi RSS and RTT-based indoor positioning. By jointly modeling spatial dependencies among APs using graph convolutions and temporal correlations across scans through causal dilated TCNs, the proposed framework achieved sub-meter accuracy across diverse indoor environments, including LOS, NLOS, and mixed conditions. Experimental results showed up to a 25% to 30% reduction in RMSE compared to existing deep and ensemble-based methods, demonstrating the model’s robustness and scalability with varying AP densities. Notably, the proposed GTCN model achieved sub-meter accuracy with as few as three to five APs in compact environments (Lecture Theatre and Office), outperforming baseline methods that require denser infrastructure or more complex ensembles to reach comparable accuracy. Moreover, increasing AP density systematically enhanced positioning accuracy, most notably in the large and structurally complex Building Floor, where the RTT-only RMSE decreased from over 9 m to below 1 m and the hybrid RSS–RTT fusion achieved 0.66 m RMSE. In contrast, compact LOS environments saturated beyond four APs, indicating that a small number of well-placed APs is sufficient when coupled with the proposed spatiotemporal learning. With fewer than $10^{5}$ trainable parameters and only tens of MFLOPs per inference, the GTCN model supports real-time edge-compatible deployment on resource-constrained devices.

Despite these promising results, this work has several limitations. First, the evaluation is restricted to four environments within a single building, with fixed AP locations and hardware; the generalization of the learned model to different buildings, floor layouts, and heterogeneous AP platforms remains to be systematically assessed. Second, the approach relies on a labeled fingerprint database per environment, and the cost of collecting and maintaining such fingerprints over long-term environmental changes (e.g., furniture rearrangements, occupancy patterns) is not explicitly addressed. Finally, RTT quality in commodity devices can be affected by vendor-specific implementations and clock behavior, which may impact positioning performance in diverse deployment settings.

Future work can be done to address these limitations along several directions. One direction is to study cross-environment and multi-floor generalization, including domain adaptation and transfer learning techniques that can reduce or eliminate the need for environment-wise training. Another is to investigate semi-supervised and self-supervised learning strategies that leverage abundant unlabeled RSS and RTT measurements to reduce the dependence on dense fingerprint surveys. Additionally, the proposed GTCN model can be extended to fuse additional modalities such as WiFi CSI and inertial measurements, enabling more robust positioning in highly dynamic or sparse WiFi scenarios. These directions aim to advance the proposed hybrid RSS and RTT GTCN framework toward the development of widely deployable and long-term stable indoor positioning systems in real-world smart buildings and IoT applications.

## Figures and Tables

**Table 1:** Overview of the WiFi RSS/RTT fingerprinting datasets used for evaluation.

Environment	Area (m^2^)	LOS Type	RPs/TPs (Train/Test)	Samples (Train/Test)
Lecture Theatre	15×14.5	LOS	88/32	5280/1920
Office	18×5.5	Mixed	81/27	4860/1620
Corridor	35×6	NLOS	85/29	5100/1740
Building Floor	92×15	Mixed	483/159	57,960/19,080

**Table 2:** Hyperparameter settings for the proposed GTCN model.

Parameter	Value
Optimizer	AdamW
Learning rate	$1\times 10^{-3}$
Batch size	64
Training epochs	1500
GCN layers	3
TCN blocks (causal)	3
Convolution kernel size	3
Dilation rates	(1, 2, 4, 8)
Hidden dimension	64
Dropout rate	0.05
Weight decay	$1\times 10^{-4}$
Huber loss parameter β	1.0
Loss weights $\left(w_{H},w_{E}\right)$	(1.0, 0.5)

**Table 3:** Positioning RMSE (m) comparison across environments using different feature combinations.

Method	Lecture Theatre	Office	Corridor	Building
JMT-SDAE [61]	0.716	0.857	0.705	1.032
RS-stacking [62]	0.724	0.824	0.672	0.967
NWEC [63]	0.663	0.781	0.599	0.965
RSS–RTT Fingerprinting [44]	0.612	0.729	0.612	0.989
RTT Fingerprinting [44]	0.559	0.718	0.704	0.988
RSS Fingerprinting [44]	2.356	1.423	1.315	1.730
Trilateration [44]	1.176	1.073	412.257	7.503
Dynamic Model [44]	0.570	0.698	0.569	0.950
Proposed (RSS-only)	2.170	1.203	1.230	1.579
Proposed (RTT-only)	0.449	0.649	0.663	0.731
Proposed (Both)	0.501	0.560	0.529	0.660

**Table 4:** Positioning performance comparison across environments and model variants.

Environment	Model	Feature Mode	RMSE (m)	Epochs
Lecture Theatre	GCN	RTT	0.426	1500
Lecture Theatre	TCN	RTT	0.410	1500
Lecture Theatre	Proposed	RTT	0.449	1500
Office	GCN	RTT	0.622	1500
Office	TCN	RTT	0.704	1500
Office	Proposed	Both	0.560	1500
Corridor	GCN	RTT	0.586	1500
Corridor	TCN	RTT	0.773	1500
Corridor	Proposed	Both	0.529	1500
Building	GCN	RTT	0.877	1500
Building	TCN	RTT	0.757	1500
Building	Proposed	Both	0.660	1500

**Table 5:** Effect of AP density on positioning performance in the Lecture Theatre.

# APs	RSS-Only	RTT-Only	RSS+RTT
3	2.337	1.929	1.364
4	2.542	0.436	0.480
5	2.170	0.449	0.501

**Table 6:** Effect of AP density on positioning performance in the Office.

# APs	RSS-Only	RTT-Only	RSS+RTT
3	1.612	0.701	0.829
4	1.417	0.713	0.687
5	1.203	0.649	0.560

**Table 7:** Effect of AP density on positioning performance in the Corridor.

# APs	RSS-Only	RTT-Only	RSS+RTT
3	1.749	0.553	0.631
4	1.230	0.663	0.529

**Table 8:** Effect of AP density on positioning performance in the Building Floor.

# APs	RSS-Only	RTT-Only	RSS+RTT
3	9.693	9.687	9.716
5	3.082	2.393	2.376
7	2.258	1.644	1.586
9	1.689	0.951	0.803
11	1.538	0.825	0.725
13	1.579	0.731	0.660

**Table 9:** Model complexity for the best configuration per environment and architecture (causal setting).

Environment	Model	Feature Mode	# Params	FLOPs (M)
Building	GCN	RSS+RTT	21,102	53.18
Building	TCN	RTT-only	81,006	26.67
Building	Proposed GTCN	RSS+RTT	93,934	67.01
Corridor	GCN	RTT-only	19,221	7.72
Corridor	TCN	RTT-only	80,853	11.58
Corridor	Proposed GTCN	RSS+RTT	93,781	17.78
Lecture Theatre	GCN	RTT-only	19,230	9.52
Lecture Theatre	TCN	RTT-only	80,862	11.83
Lecture Theatre	Proposed GTCN	RTT-only	90,910	17.86
Office	GCN	RTT-only	19,230	9.52
Office	TCN	RTT-only	80,862	11.83
Office	Proposed GTCN	RSS+RTT	93,790	19.59

## Data Availability

All the datasets used for this work are publicly available in https://github.com/Fx386483710/WiFi_RSS_RTT_Dataset_for_Model_Selection/tree/master?tab=readme-ov-file (accessed on 8 August 2025).

## Abbreviations

The following abbreviations are used in this manuscript:

NLOS	Non-Line-of-Sight
NLOS	Line-of-Sight
GTCN	Graph–Temporal Convolutional Network
AP	Access Point
RP	Reference Point
RSS	Received Signal Strength
RTT	Round Trip Time
CSI	Channel State Information
TCN	Temporal Convolutional Networks
CDF	Cumulative Distribution Function
RMSE	Root Mean Square Error
